# Data on the presence or absence of genes encoding essential proteins for ochratoxin and fumonisin biosynthesis in *Aspergillus niger* and *Aspergillus welwitschiae*

**DOI:** 10.1016/j.dib.2016.03.016

**Published:** 2016-03-10

**Authors:** Fernanda Pelisson Massi, Daniele Sartori, Larissa de Souza Ferranti, Beatriz Thie Iamanaka, Marta Hiromi Taniwaki, Maria Lucia Carneiro Vieira, Maria Helena Pelegrinelli Fungaro

**Affiliations:** a Centro de Ciências Biológicas, Universidade Estadual de Londrina, P.O. Box 6001, Londrina 86051-990, Brazil; b Instituto de Tecnologia de Alimentos, P.O. Box 139, Campinas 13070-178, Brazil; c Departamento de Genética, Escola Superior de Agricultura “Luiz de Queiroz” USP, P.O. Box 83, Piracicaba 13418-900, Brazil

**Keywords:** Ochratoxin, Fumonisin, High-resolution capillary electrophoresis

## Abstract

We present the multiplex PCR data for the presence/absence of genes involved in OTA and FB_2_ biosynthesis in *Aspergillus niger*/*Aspergillus welwitschiae* strains isolated from different food substrates in Brazil. Among the 175 strains analyzed, four mPCR profiles were found: Profile 1 (17%) highlights strains harboring in their genome the *pks*, *radH* and the *fum8* genes. Profile 2 (3.5%) highlights strains harboring genes involved in OTA biosynthesis i.e. *radH* and *pks*. Profile 3 (51.5%) highlights strains harboring the *fum8* gene. Profile 4 (28%) highlights strains not carrying the genes studied herein. This research content is supplemental to our original research article, “Prospecting for the incidence of genes involved in ochratoxin and fumonisin biosynthesis in Brazilian strains of *A. niger* and *A. welwitschiae*” [1].

## Specifications table

**Table:** 

Subject area	Microbiology
More specific subject area	Mycology
Type of data	Figure
How data was acquired	Automated high-resolution capillary electrophoresis (CE). ABI 3500XL Genetic Analyser (Applied Biosystems, USA)
Data format	Analyzed
Experimental factors	A total of 175 strains of *Aspergillus niger* and *Aspergillus welwitschiae* isolated from different food substrates in distinct geographical regions of Brazil were submitted to DNA extraction and multiplex PCR analysis
Experimental features	Multiplex PCR using four primer pairs in one amplification reaction was carried out as described by Massi et al. [1]. The amplified products were analyzed using standard capillary electrophoresis
Data source location	Brazil
Data accessibility	The data is with this article

## Value of the data

• The methodology presented here is potentially valuable to other researchers for developing similar assays for studying multiple genes simultaneously.
• Particularly, the multiplex PCR as presented here is useful to survey for the occurrence of *A. niger*/*A. welwitschiae* strains harboring essential genes for ochratoxin and fumonisin biosynthesis.
• The frequency of strains of *A. niger*/*A. welwitschiae* harboring essential genes for ochratoxin and fumonisin biosynthesis could be compared to that obtained from other countries.
• The multiplex PCR here developed is relevant to evidence specific non-producing mycotoxin phenotypes.

## 1 Data

**Fig. 1:**
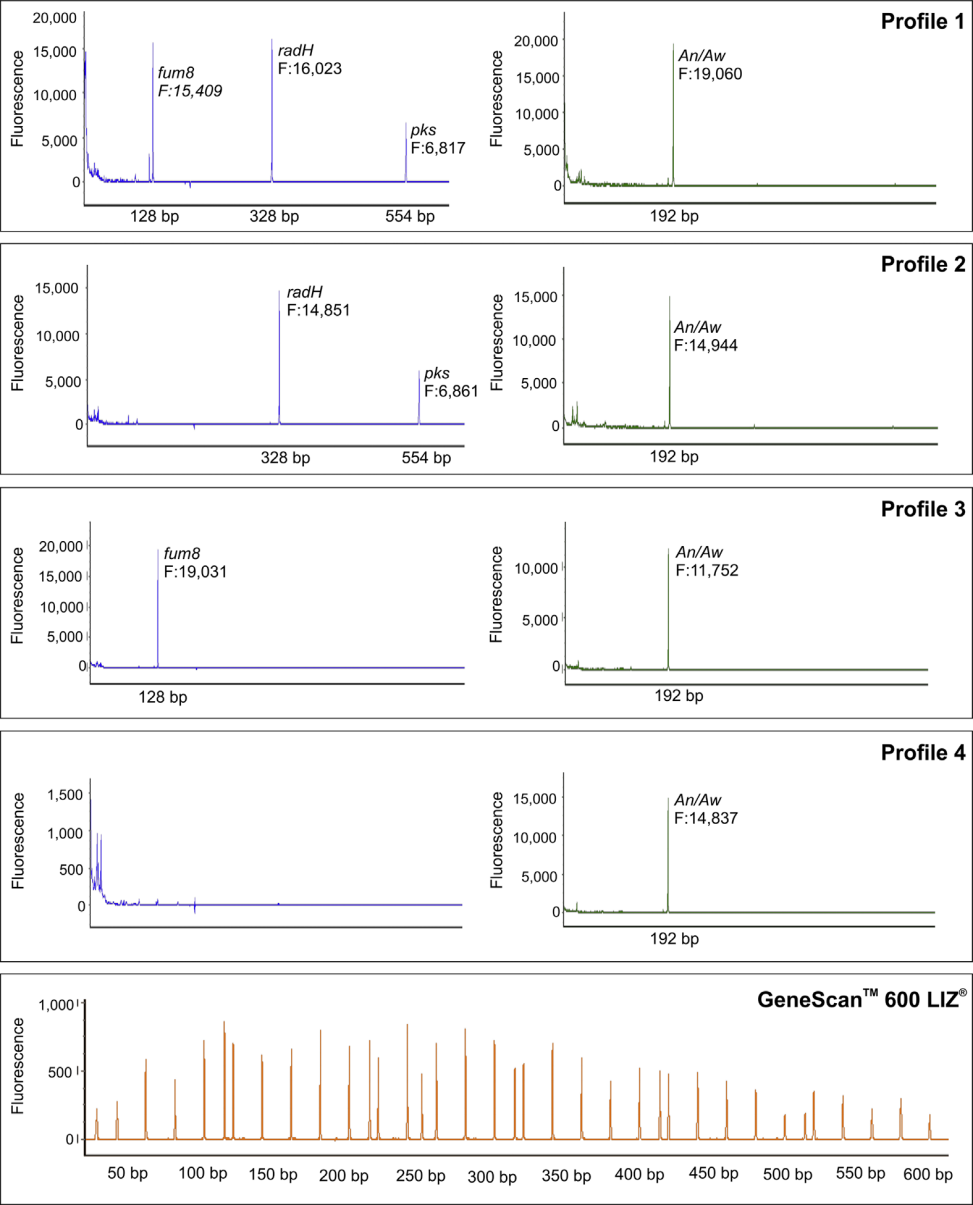
Genotype profiles revealed by the ABI 3500XL Genetic Analyzer with GeneMarker® Software. Four PCR products were amplified with primers labeled with FAM (blue) and HEX (green). Profile 1 indicates that the strain is *A. niger* or *A. welwitschiae* (green, 192 bp) harboring the *pks* (blue, 554 bp), *radH* (blue, 328 bp) and the *fum8* (blue, 128 bp) genes. Profile 2 indicates that the strain is *A. niger* or *A. welwitschiae* harboring the genes involved in OTA biosynthesis i.e. *radH* and *pks*. Profile 3 indicates that the strain is *A. niger* or *A. welwitschiae* harboring the gene *fum8*. Profile 4 indicates that the strain is *A. niger* or *A. welwitschiae* harboring none of genes studied herein.

Among 175 *A. niger*/*A. welwitschiae* strains analyzed, we found four mPCR profiles (Fig. 1). Profile 1 (17%) highlights strains harboring the *pks* (shown in blue, 554 bp), *radH* (blue, 328 bp) and *fum8* (blue, 128 bp) genes. Profile 2 (3.5%) highlights strains harboring only genes involved in OTA biosynthesis (*radH* and *pks*). Profile 3 (51.5%) highlights strains harboring only the gene *fum8*. Profile 4 (28%) highlights strains not carrying the mycotoxigenic genes studied herein.

## 2 Experimental design, materials and methods

**Fig. 2:**
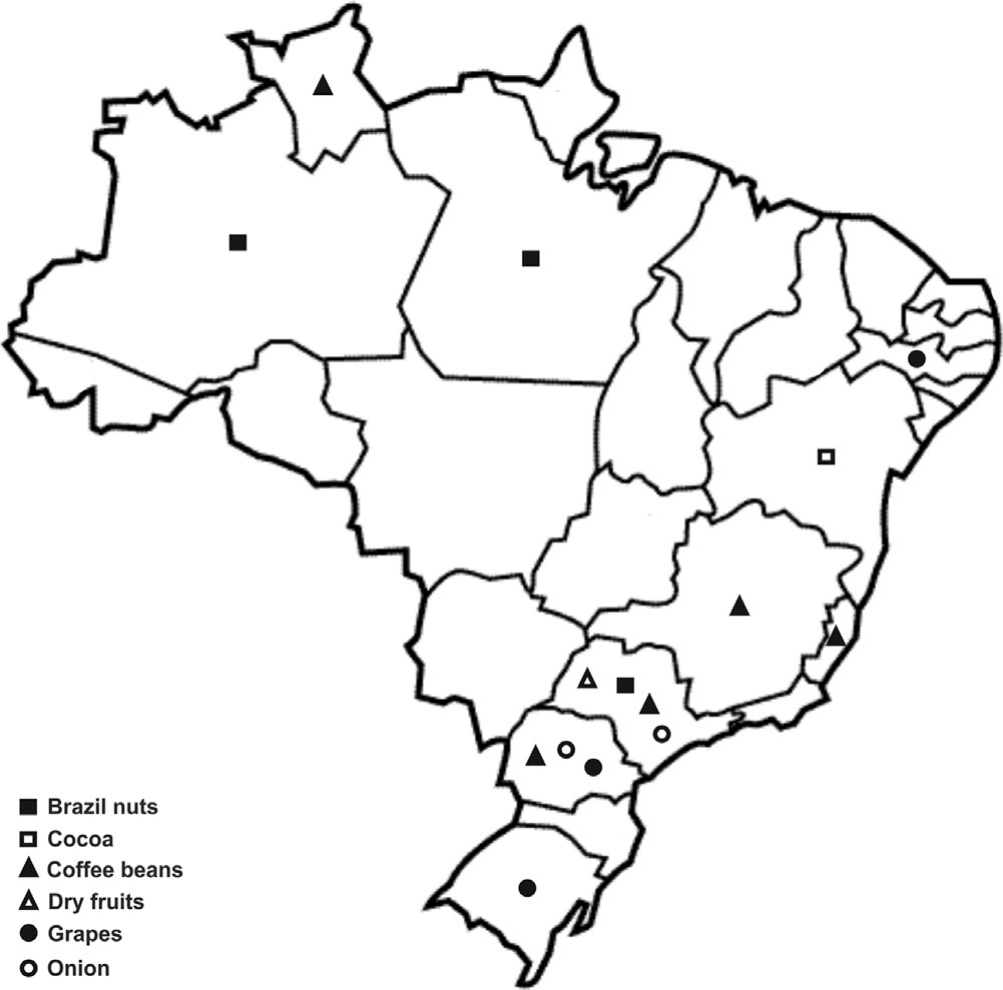
Brazilian map depicting the locations from which samples were obtained.

We used a set of primer-pairs to survey for the presence/absence of genes involved in OTA and FB_2_ biosynthesis in *A. niger*/*A. welwitschiae* strains [1], which were collected from dried fruits (*n*=19), Brazil nuts (*n*=30), coffee beans (*n*=27), grapes (*n*=40), cocoa (*n*=3), and onions (*n*=56). The Brazilian geographical regions where the samples were collected are shown in Fig. 2. The mycotoxigenic genes investigated herein were those encoding a polyketide synthase (*pks*), a flavin-dependent halogenase (*radH*), both involved in ochratoxin biosynthesis, and a α-oxoamine synthase (*fum8*), essential for fumonisin biosynthesis. A pair of *A. niger*/*A. welwitschiae*-specific primers targeting the β-tubulin gene (*benA*) was also included in the amplification reaction. Multiplex amplifications (*m*PCR) were carried out using four primer-pairs in a single reaction mixture, as described by Massi et al. [1]. Each amplified sample was diluted 10× and 8.0 µL of (Hi-Di) formamide and 0.3 µL of GeneScan™ 600 LIZ^®^ internal lane size standard (Applied Biosystems, USA) were added to 2 µL of the diluted sample. An ABI 3500XL Genetic Analyzer (Applied Biosystems, USA) was used to separate and detect the fluorescently labeled PCR products which were analyzed using GeneMarker^®^ Software (SoftGenetics^®^).
