# Supplementary material for: Data on the presence or absence of genes encoding essential proteins for ochratoxin and fumonisin biosynthesis in Aspergillus niger and Aspergillus welwitschiae
Source: Data Brief. 2016 Mar 10;7:704–8. doi: 10.1016/j.dib.2016.03.016 (PMC4802528; doi:10.1016/j.dib.2016.03.016)
Supplement: Supplementary file 1 — Supplementary material [file mmc1.pdf]

Conflicts of Interest Statement

Manuscript title: Data on the presence or absence of genes encoding essential proteins for ochratoxin and fumonisin biosynthesis in Aspergillus niger and Aspergillus welwitschiae

The authors whose names are listed immediately below certify that they have NO affiliations with or involvement in any organization or entity with any financial interest (such as honoraria; educational grants; participation in speakers' bureaus; membership, employment, consultancies, stock ownership, or other equity interest; and expert testimony or patent-licensing arrangements), or non-financial interest (such as personal or professional relationships, affiliations, knowledge or beliefs) in the subject matter or materials discussed in this manuscript.

Author names: Fernanda Pelisson Massi
Daniele Sartori
Larissa de Souza Ferranti
Beatriz Thie Iamanaka
Marta Hiromi Taniwaki
Maria Helena P. Fungaro
Maria Lucia Carneiro Vieira

The authors whose names are listed immediately below report the following details of affiliation or involvement in an organization or entity with a financial or non-financial interest in the subject matter or materials discussed in this manuscript. Please specify the nature of the conflict on a separate sheet of paper if the space below is inadequate.

Author names:

This statement is signed by all the authors to indicate agreement that the above information is true and correct (*a photocopy of this form may be used if there are more than 10 authors*):

Author's name (typed)	Author's signature	Date
Fernando Pelisson Massi	[illegible]	
Daniele Sartori	Daniele Sartori	
Larissa de Souza Ferranti	Larissa Ferranti	
Beatriz Thie Iamanaka	Beatriz Thie Iamanaka	
Marta Hiromi Taniwaki	Marta H. Taniwaki	
Maria Lucia Carneiro Vieira	Maria Lucia Carneiro Vieira	17.2.2016
Maria Helena P. Fungaro	[illegible]	
